# Indigenous use and bio-efficacy of medicinal plants in the Rasuwa District, Central Nepal

**DOI:** 10.1186/1746-4269-6-3

**Published:** 2010-01-26

**Authors:** Yadav Uprety, Hugo Asselin, Emmanuel K Boon, Saroj Yadav, Krishna K Shrestha

**Affiliations:** 1 Human Ecology Department, Vrije Universiteit Brussel, Laarbeeklaan 109, B-1090 Brussels, Belgium; 2 Canada Research Chair in Aboriginal Forestry, Université du Québec en Abitibi-Témiscamingue, 445, boulevard de l'Université, Rouyn-Noranda, Québec, J9X 5E4, Canada; 3 Central Department of Botany, Tribhuvan University, Kathmandu, Nepal; 4 Canada Research Chair in Aboriginal Forestry, Université du Québec en Abitibi-Témiscamingue, 445, boulevard de l'Université, Rouyn-Noranda, Québec, J9X 5E4, Canada

## Abstract

**Background:**

By revealing historical and present plant use, ethnobotany contributes to drug discovery and socioeconomic development. Nepal is a natural storehouse of medicinal plants. Although several ethnobotanical studies were conducted in the country, many areas remain unexplored. Furthermore, few studies have compared indigenous plant use with reported phytochemical and pharmacological properties.

**Methods:**

Ethnopharmacological data was collected in the Rasuwa district of Central Nepal by conducting interviews and focus group discussions with local people. The informant consensus factor (F_IC_) was calculated in order to estimate use variability of medicinal plants. Bio-efficacy was assessed by comparing indigenous plant use with phytochemical and pharmacological properties determined from a review of the available literature. Criteria were used to identify high priority medicinal plant species.

**Results:**

A total of 60 medicinal formulations from 56 plant species were documented. Medicinal plants were used to treat various diseases and disorders, with the highest number of species being used for gastro-intestinal problems, followed by fever and headache. Herbs were the primary source of medicinal plants (57% of the species), followed by trees (23%). The average F_IC_ value for all ailment categories was 0.82, indicating a high level of informant agreement compared to similar studies conducted elsewhere. High F_IC _values were obtained for ophthalmological problems, tooth ache, kidney problems, and menstrual disorders, indicating that the species traditionally used to treat these ailments are worth searching for bioactive compounds: *Astilbe rivularis*, *Berberis asiatica*, *Hippophae salicifolia, Juniperus recurva*, and *Swertia multicaulis*. A 90% correspondence was found between local plant use and reported plant chemical composition and pharmacological properties for the 30 species for which information was available. Sixteen medicinal plants were ranked as priority species, 13 of which having also been prioritized in a country-wide governmental classification.

**Conclusions:**

The *Tamang *people possess rich ethnopharmacological knowledge. This study allowed to identify many high value and high priority medicinal plant species, indicating high potential for economic development through sustainable collection and trade.

## Background

Ethnobotany reveals historical and present plant use to fulfil a wide variety of human needs [1,2]. Documenting indigenous knowledge through the ethnobiological approach is important for species conservation and sustainable resource use [3]. Furthermore, such studies are often significant in revealing locally important plant species, sometimes leading to the discovery of crude drugs [4,5], or contributing to economic development.

Globally, millions of people in the developing world rely on medicinal plants for primary health care, income generation and livelihood improvement [6]. Indigenous people living on their traditional territory largely rely on medicinal plants for healthcare and they are therefore rich in ethnopharmacological knowledge. The interest in phytomedicine has been renewed over the last decade and several medicinal plant species are now being screened for pharmacological potential. According to Laird and Pierce [7], the world market for herbal remedies was worth 19.4 billion US$ in 1999. The global demand for medicinal plants is increasing and, in India alone, the market is expanding at an annual rate of 20% [8,9]. Scientific research is needed to determine the active principles of traditional medicinal recipes and to evaluate their effectiveness, so that benefits could be equally shared among local peoples in the spirit of the Convention on Biological Diversity [10].

Medicinal plants play vital roles in the Nepalese livelihood [11] and the use of medicinal plants is frequent in several Nepalese regions [12-14]. It is estimated that only 15-20% of the population of Nepal - living in and around urban areas - have access to modern medicinal facilities, whereas the rest depend on traditional medicines [11]. Nepal is a natural storehouse of medicinal plants [12,15,16]. Each year thousands of tons of raw material are exported, mostly to India, but also to Asia, Europe and America [17]. The government of Nepal aims to promote medicinal plant use and conservation programmes for livelihood improvement and poverty alleviation through various policies [11]. However, the contribution of this sector to the national economy is still nominal.

Several ethnopharmacological studies have been conducted in Nepal [13,15,16,18,19], but many parts of the country remain unexplored. Few studies have attempted to estimate use variability of Nepalese medicinal plants or to evaluate their bio-efficacy [20,21]. Therefore, this study was conducted in order to achieve the following objectives:

1. Document the medicinal plants used in the traditional healthcare delivery system of the Chilime Village Development Committee (VDC) of the Rasuwa district of Central Nepal,

2. Estimate use variability of medicinal plants, indicating informant agreement,

3. Evaluate the bio-efficacy of medicinal plants by comparing local use with findings from published phytochemical and pharmacological studies,

4. Identify priority medicinal plant species for the Rasuwa district.

## Study area

**Figure 1:**
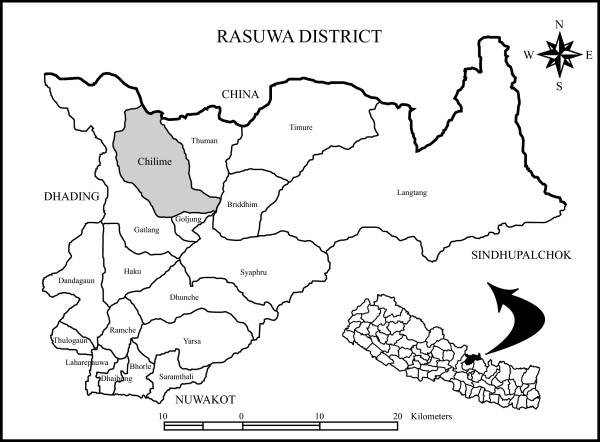
**Location of the study area in the Rasuwa district of Central Nepal**.

A field study was carried out in the Chilime VDC of the Rasuwa district of Central Nepal. The district lies between 27° 2' and 27° 10' N and 84° 45' and 85° 88' E, with altitude ranging from 792 to 7245 m a.s.l. The Rasuwa district presents some of the best examples of graded climatic conditions in Central Himalaya. Pronounced altitudinal gradients, coupled with complex topography and geology, have resulted in a rich biodiversity and unique vegetation patchwork [22]. Therefore, the district harbours a rich diversity of medicinal plants. The Chilime VDC lies in the northern part of the district, bordering the Tibetan part of China, and comprises temperate to alpine climates within 2000-4700 m altitude (Fig. 1). The local inhabitants are part of the *Tamang *indigenous people, which comprises 98% of the total Chilime VDC population [23]. People from the *Tamang *ethnic group have a rich culture and possess sound traditional knowledge. However, they are economically and socially marginalized and far from having their basic needs fulfilled.

## Methods

Ethnopharmacological data was collected by conducting interviews and focus group discussions with local people (50% > 40 years old), from July to September 2007. A total of 50 household heads (56% male and 44% female) from the Chilime VDC of the Rasuwa district participated in the study. Participants were purposively selected to include key informants [24] like plant collectors, medicinal plant cultivators, traditional healers, and traders. Respondents were all from the *Tamang *ethnic group, predominant (65%) in the Rasuwa district. The majority (62%) of the respondents had no formal education, 18% had primary school education, 12% had secondary education, and 8% had university level education. Prior informed consent was obtained with the help of community workers [25] that also facilitated interviews and discussions with the local people. Consent was granted by the local people for the dissemination of their traditional knowledge.

Guidelines for the interviews and group discussions were developed to facilitate the collection of information. Interviews and group discussions were conducted to gather information on plant uses, parts used, and modes of utilization. A checklist was developed and used to determine what species were used to treat what kinds of diseases/disorders. Herbarium specimens were collected for those species for which field identification was not certain and brought back to the lab to facilitate identification using reference collections [26-29] and expert knowledge. The specimens are deposited at the Tribhuvan University Central Herbarium (TUCH).

The informant consensus factor (F_IC_) was calculated to estimate use variability of medicinal plants [30,31]. F_IC _values range from 0.00 to 1.00. High F_IC _values are obtained when only one or a few plant species are reported to be used by a high proportion of informants to treat a particular ailment, whereas low F_IC _values indicate that informants disagree over which plant to use [30]. High F_IC _values can thus be used to pinpoint particularly interesting species for the search of bioactive compounds [31]. F_IC _is calculated using the following formula [30,31]:

where N_ur _is the number of individual plant use reports for a particular illness category, and N_t _is the total number of species used by all informants for this illness category.

Medicinal plant species were ranked according to prioritization criteria developed by the Herbs and Non-Timber Forest Products Coordination Committee of Nepal and the National Medicinal Plants Board of India, and synthesized at the First National Trade Show and Seminar on Herbs, Herbal Products and Spices, held November 12-14, 2005 in Nepalgunj, West Nepal [32]:

• Market value/price

• Quantity exported annually recorded by the District Forest Office

• Average annual export to India and abroad

• Annual industrial demand in Kathmandu

• Ease of cultivation/domestication

• Royalties

• Parts used

• Bulkiness (availability in large quantities)

• Social acceptance for further processing

• Quality improvement

• Distribution range

• Threat category

• Legal protection

• Availability of local processing techniques

• Regeneration/rotation period

• Ethno-botanic importance

• Potential for further processing

Criteria accounting for availability, local knowledge and use, and market demand were given more weight. Indigenous uses determined from interviews and discussion groups, and phytochemical and pharmacological properties determined from a review of the available literature were compared for all species for which information was available.

## Results

**Figure 2:**
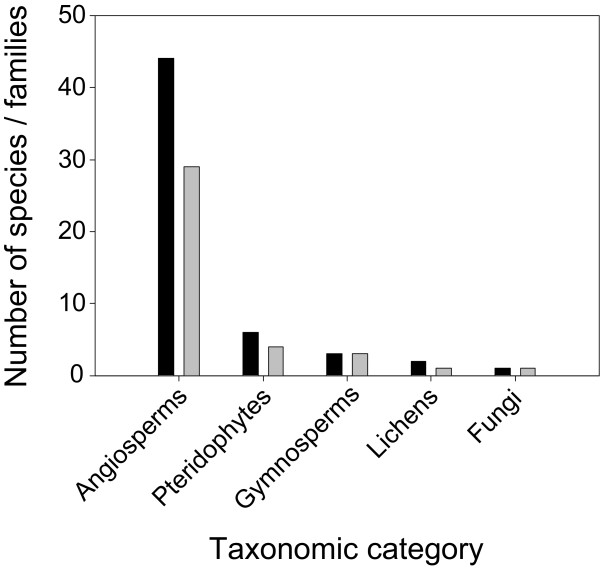
**Number of medicinal plant species (black bars) and families (grey bars) in major taxonomic categories**.

**Figure 3:**
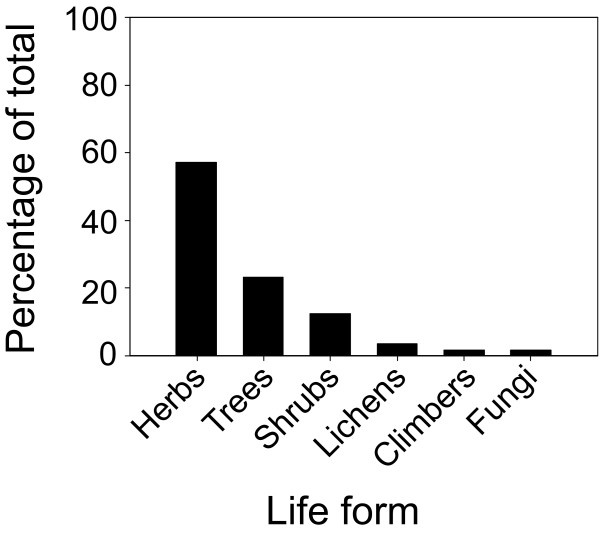
**Percentage distribution of medicinal plant species according to life form**.

The ethnobotanical survey identified a total of 56 medicinal plant species used to prepare a wide variety of remedies (Additional file 1). Angiosperms were predominant, with 44 species belonging to 29 families (Fig. 2), followed by Pteridophytes (6 species from 4 families), Gymnosperms (3 species from 3 families), Lichens (2 species from 1 family) and Fungi (1 species). The prevailing life form was herbs, followed by trees, shrubs, lichens, climbers and fungi (Fig. 3).

**Figure 4:**
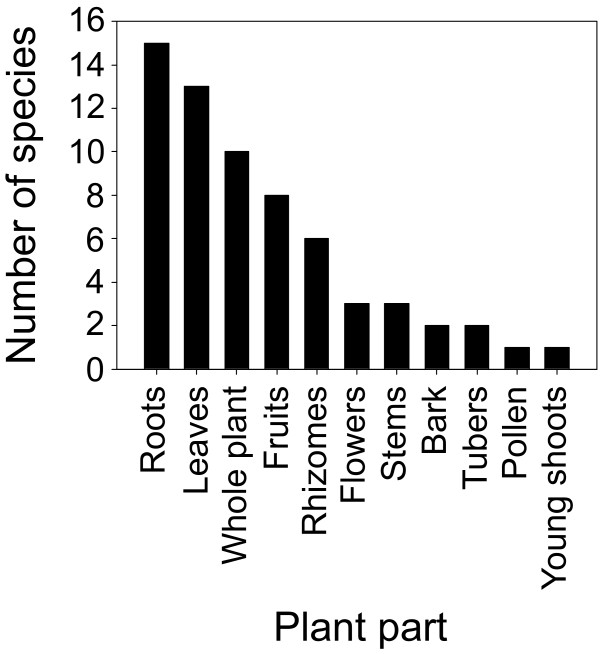
**Use frequency (number of species) of different plant parts in traditional medicine preparation**.

Almost all plant parts were used to prepare different medicinal formulations: roots, rhizomes, tubers, bark, leaves, flowers, fruits, pollen, young shoots, and whole plants (Additional file 1). The most frequently used plant parts were roots, followed by leaves, whole plants, fruits, and rhizomes (Fig. 4). Use of multiple plant parts was also recorded in a few cases (Additional file 1).

### Ailments treated and remedies formulation

Cuts and wounds, respiratory problems, gastro-intestinal disorders, cough and cold, musculo-skeletal problems, fever and headache, weakness and dizziness, menstrual disorders, dermatological infections, ophthalmological problems, and tooth ache were the main ailments treated with medicinal plants. Gastro-intestinal disorders, fever and headache, cuts and wounds, cough and cold, and musculo-skeletal problems were treated with the highest diversity of medicinal plant species (Additional file 1, Table 1). Although most species were only used to treat one ailment (34/56), some were found having up to four different medicinal uses (Table 1).

**Table 1:** Medicinal plants used to cure various ailments.

Ailment	Plants
Gastro-intestinal disorders	***Asparagus racemosus, Berginia ciliata***, *Bistorta affinis, Cannabis sativa, Cheilanthes albomarginata*, ***Dactylorhiza hatagirea***, *Fritillaria cirrhosa, Hippophae salicifolia, Hippophae tibetana, Lepisorus mehrae, Lindera nessiana, Mahonia napaulensis*, ***Paris polyphylla***, *Potentilla fulgens*, ***Primula sikimmensis, Rheum australe, Rhodiola himalensis***, *Rhododendron anthopogon, Vitex negundo, Zanthoxylum armatum*

Fever and headache	***Aconitum spicatum, Asparagus racemosus, Berberis asiatica, Bergenia ciliata, Delphinium himalayai***, *Drynaria propinqua*, ***Geranium nepalense***, *Juniperus recurva, Lonicera myrtillus*, ***Nardostachys grandiflora, Onychium japonicum, Paris polyphylla***, *Pieris formosa*, ***Primula sikkimmensis, Rheum australe, Rhodiola himalensis, Swertia chirayita***, *Swertia multicaulis*

Cuts and wounds	***Aconitum spicatum***, *Amaranthus spinosus, Artemisia indica*, ***Dactylorhiza hatagirea***, *Eupatorium adenophorum*, ***Geranium nepalense***, *Lycopodium clavatum*, ***Lyonia ovalifolia***, *Parmelia cirrhata, Parmelia *sp., ***Valeriana jatamansi***

Cough and cold	***Abies spectabilis***, *Acorus calamus*, ***Anaphalis contorata, Delphinium himalayai***, *Hippophae salicifolia, Juniperus recurva*, ***Swertia chirayita***, *Swertia multicaulis*, ***Valeriana jatamansi***

Musculo-skeletal problems	*Aconitum ferox, Entada rheedei, Fraxinus floribunda, Neopicrorhiza scrophulariiflora*, ***Phymatopteris quasidivaricata***, ***Valeriana jatamansi***

Respiratory problems	***Abies spectabilis***, *Ephedra gerardiana, Taxus wallichiana*, ***Valeriana jatamansi***

Weakness and dizziness	*Cordyceps sinensis, Juglans regia*, ***Nardostachys grandiflora***, *Rhododendron arboretum*

Dermatological infections	***Lyonia ovalifolia, Onychium japonicum, Phymatopteris quasidivaricata***, *Rubia manjith*

Menstrual disorders	*Astilbe rivularis*, *Hippophae salicifolia*

Ophthalmological problems	***Berberis asiatica***

Tooth ache	*Swertia multicaulis*

Kidney problems	*Juniperus recurva*

Others	***Anaphalis contorata***, *Myrica esculenta*

Bold: species used to treat two different ailments (18/56). Underlined: species used to treat three different ailments (3/56). Bold and underlined: species used to treat four different ailments (1/56).

Most people who participated in interviews and group discussions were familiar with the species used to deal with common ailments like cough and cold, digestive problems, fever, headache, skin infection, and in such cases plant based medicinal remedies were used on a regular basis. For complex problems like chest pain, menstrual disorders, rheumatism, or eye and kidney problems, people took advice from local traditional healers. Traditional healers believe in a form of sanctity of the curative power of medicinal plants. They thus keep secrecy over remedy formulation, believing that the medicines would lose their potency if revealed to other people.

**Figure 5:**
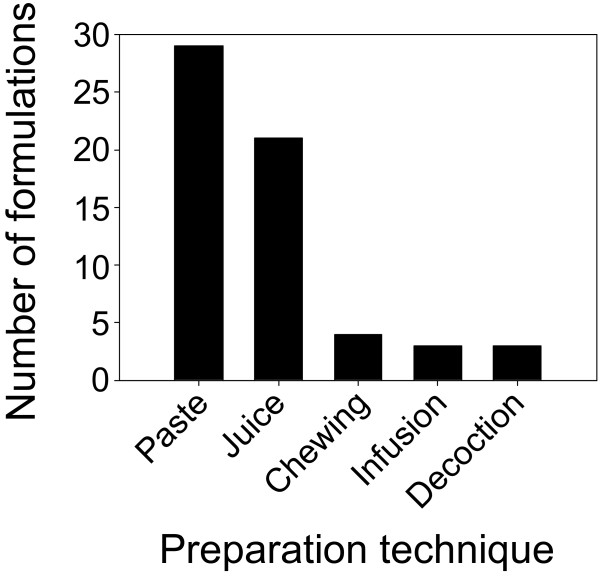
**Use frequency (number of medicinal formulations) of different remedy preparation techniques**.

A total of 60 medicinal formulations were prepared from the 56 medicinal plants identified in this study. Two formulations were prepared using five different species, while all other formulations were prepared using a single species. Preparation methods included paste, juice, decoction, infusion and chewing the raw plant (Table 2, Fig. 5). The majority of formulations were prepared as paste or juice. Crushing, pounding, and grinding are executed using a pestle in a mortar made of hard stone.

**Table 2:** Common forms of preparation methods for remedies made of medicinal plants.

Preparation method	Description
Paste	Fresh plant parts are crushed with a stone pestle and mortar.
Juice	Obtained by squeezing or crushing plant parts and filtering through cloth. Sometimes requires addition of freshwater or other liquid for dilution.
Chewing	Fresh plant parts are chewed.
Infusion	Plant parts are plunged in water for a few minutes.
Decoction	Plant parts are boiled in water and the extract (crude drug) is used.

### Informant consensus factor

The level of informant agreement was high for most ailment categories (mean F_IC _= 0.82) and total consensus (F_IC _= 1.00) was even obtained for ophthalmological problems, tooth ache and kidney problems (Table 3). Gastro-intestinal disorders, as well as fever and headache showed relatively low levels of consensus (F_IC _= 0.53 and 0.61, respectively).

**Table 3:** Informant consensus factor (F_IC_) for different ailment categories.

Ailment	Number of taxa (N_t_)	Number of use reports (N_ur_)	Informant consensus factor (F_IC_)
Gastro-intestinal ailments	20	41	0.53

Fever and headache	18	45	0.61

Musculo-skeletal problems	6	16	0.67

Weakness and dizziness	4	10	0.67

Cuts and wounds	11	48	0.79

Cough and cold	9	44	0.81

Respiratory problems	4	26	0.88

Dermatological infections	4	39	0.92

Menstrual disorders	2	21	0.95

Ophthalmological problems	1	5	1.00

Tooth ache	1	10	1.00

Kidney problems	1	2	1.00

Total	81*	307	-

*Some taxa were reported in more than one ailment category (see Table 2).

### Prioritization of medicinal plant species

Respondents were asked to prioritize medicinal plant species based on the criteria developed at the First National Trade Show and Seminar on Herbs, Herbal Products and Spices (see Methods and [32]). After consultations with the local people, district forest staff, NGO representatives, and researchers, a final list of 16 high-priority species was obtained (Table 4).

**Table 4:** List of priority medicinal plant species for the Rasuwa district of Central Nepal.

Rank	Prioritization score (/50)	Species name
1	46	*Nardostachys grandiflora *DC.
2	45	*Parmelia *spp.
3	44	*Swertia chirayita *(Roxb. ex Fleming) Karsten
4	44	*Aconitum spicatum *(Bruhl) Stapf
5	44	*Delphinium himalayai *Munz
6	41	*Neopicrorhiza scrophulariiflora *(Pennell) D.Y. Hong
7	38	*Rheum australe *D. Don
8	36	*Fritillaria cirrhosa *D. Don
9	36	*Dactylorhiza hatagirea *(D. Don) Soo
10	35	*Valeriana jatamansii *Jones
11	34	*Taxus wallichiana *Zucc
12	32	*Zanthoxylum armatum *DC
13	28	*Bergenia ciliata *(Haw.) Sternb
14	24	*Paris polyphylla *Sm.
15	22	*Acorus calamus *L.
16	20	*Asparagus racemosus *Willd.

### Bio-efficacy of traditionally-used medicinal plants

Phytochemical and pharmacological studies were found in the literature for 30 of the 56 medicinal plant species used by the *Tamang *people of the Chilime VDC, Rasuwa district, Central Nepal. Comparison of reported traditional use with known phytochemical and pharmacological properties showed complete or partial correspondence for 27 of the 30 plants (Table 5). Twelve of the 30 plants for which phytochemical/pharmacological information was found in the literature were high-priority species in the Rasuwa district (Tables 4 and 5).

**Table 5:** Comparison of local use and phytochemical/pharmacological properties of medicinal plants.

Species	Indigenous use (Present study)	Phytochemical/pharmacological properties (Literature review)	Local use coherent with known phytochemical/pharmacological properties
*Aconitum ferox*	Root paste is taken for joint pain.	Alkaloid extract may possess anti-inflammatory properties [48].	Yes

Acorus calamus*	Rhizome is used for cough/cold, and throat pain.	Antimicrobial properties [49].	Yes

*Amaranthus spinosus*	Root paste is applied on cuts and wounds.	Contains several chemical compounds, including tannins (coagulant), steroids (muscle building), flavonoids (antimicrobial), and volatile oils (antiseptic) [50].	Yes

*Artemisia indica*	Leaf paste is applied on cuts and wounds.	Antimicrobial properties [49].	Yes

Asparagus racemosus*	Tuber paste is used for fever, stomach ache, and diarrhoea.	Ethanol and aqueous extracts from the tubers exhibit significant antidiarrheic activity [51].	Yes

*Berberis asiatica*	Cambium paste is used for rheumatism and pith paste is used for eye problems.	Widespread use as an extract in eye drops for conjunctivitis [21]. Effective as an antipyretic, anaesthetic, and antihypertensive [52].	Yes

Bergenia ciliata*	Whole plant juice is taken to treat indigestion, fever, diarrhoea, and dysentery.	Plants possess antipyretic, antidiarrheic, diuretic and expectorant properties [21].	Yes

*Cannabis sativa*	Plant paste is taken for stomach problems.	Diuretic, anti-emetic, anti-epileptic, painkilling, anti-inflammatory, and antipyretic properties [53].	Yes

*Cordyceps sinensis*	Whole plant juice is taken as a tonic.	Largely recognised as inducing sexual power and vitality [16,54,55].	Yes

*Eupatorium adenophorum*	Leaf juice is applied on cuts and wounds.	Contains haemostatic ayapanin [20].	Yes

*Fraxinus floribunda*	Bark infusion is used for body pain.	Anti-inflammatory, anti-oxidative and skin regenerating activities [56].	Yes

Fritillaria cirrhosa*	Plant juice is taken for stomach disorders.	Plant contains steroidal alkaloids effective against stomach disorders [57].	Yes

*Hippophae salicifolia*	Fruit juice is taken for cough, diarrhoea, and menstrual disorder.	Contains high levels of flavonoids (with antimicrobial properties and effectiveness against menopausal symptoms), carotenoids and vitamin C [58].	Yes

*Hippophae tibetana*	Fruit juice is taken for stomach disorders.	Contains high levels of flavonoids (antimicrobial), carotenoids and vitamin C [58].	Yes

*Juglans regia*	Fruit juice is taken as a tonic.	Seeds are diuretic and a nervous system depressant [59].	No

*Lindera neesiana*	Fruit juice taken for diarrhoea.	Essential oil extracted from fruits possess significant antimicrobial activity [60].	Yes

*Lycopodium clavatum*	Pollen paste is used on cuts and wounds.	Contains anti-inflammatory alkaloidal-types of compounds [61].	Yes

Nardostachys grandiflora*	Whole plant juice is taken to treat headache and high altitude sickness.	Ethanol extract from roots showed anticonvulsant activity and are a nervous system stimulant [62].	Partial

Neopicrorhiza scrophulariiflora*	Used for body pain.	Contains glycosides [63].	Unknown

*Onychium japonicum*	Used for skin problems.	Onychin-a flavanone glycoside is cytotoxic [64].	No

Paris polyphylla*	Root paste is taken for fever, vomiting and worms.	A methanolic extract is gastroprotective [65]. Also possesses anthelmintic properties [66].	Yes

*Potentilla fulgens*	Root paste is used against gastritis.	Possess antibacterial and anti-inflammatory properties [67].	Yes

Rheum australe*	Root juice is taken for fever, indigestion, diarrhoea, and stomach ache.	Purgative, astringent, and anti-amoebic effects [68].	Yes

*Rhododendron anthopogon*	Flower is chewed for stomach ache.	Volatile components possess antimicrobial activities [69].	Yes

*Rubia manjith*	Root paste is applied over scabies and other skin diseases.	Anti-proliferative against epidermal keratinocytes [70]. Antiseptic properties [16,71].	Yes

Swertia chirayita*	Whole plant juice is used for fever, cold and headache.	An aqueous extract is antipyretic [72], and an ethanolic extract is antibacterial and antifungal [73]. An aqueous extract is anti-inflammatory [74].	Yes

Taxus wallichiania*	Leaf juice is drunk to treat respiratory problems.	Antimicrobial effect [75].	Yes

Valeriana jatamansi*	Rhizome paste is applied on cuts and wounds and joint problems. Rhizome is chewed to treat throat pain.	Analgesic, carminative, antispasmodic, antiseptic, expectorant, diuretic and sedative properties [76].	Yes

*Vitex negundo*	Seed paste is used for worms.	Possesses antifeedant, antibacterial and anti-inflammatory properties [77,78].	Yes

Zanthoxylum armatum*	Fruits are crushed, pickled and taken for stomach ache and indigestion.	Ethanol fruit extract is antibacterial against gram positive bacteria (*Bacillus subtilis*, *Staphylococcus aureus*, *Mycobacterium phlei*) [79].	Yes

Species identified by an asterisk are high-priority species of the Rasuwa district (see Table 4).

## Discussion

### Traditional use of medicinal plants in Chilime

Altogether, 56 species of medicinal plants were identified as being used in traditional medical systems in the Rasuwa district of central Nepal. As indicated for the Dolkha district, having more or less the same economic, social and ecological characteristics, reliance on medicinal plants for health care was associated with poverty, lack of accessibility to modern healthcare facilities, and traditional belief about plant effectiveness [20]. Herbs are the primary source of medicinal plant species, followed by trees, most likely because herbs are more abundant. It is believed that the more abundant a plant is, the more medicinal virtues it may possess [20,33]. The ease with which plants can be collected, stored, and transported and the ease with which bioactive compounds can be extracted are also factors that contribute to the preference for herbs [20]. Moreover, most species used in the traditional health care system of the Chilime VDC are harvested from the wild. This is common practice all over the world, as was observed in Cameroon [34], Uganda [35] and Peru [36], for example. The preference for root to prepare traditional remedies follows the scientific reasoning that roots generally contain high concentrations of bioactive compounds [37].

### Informant agreement

The average F_IC _value for all ailment categories was 0.82, indicating a high level of informant agreement compared to similar studies conducted in Mexico [30,31,38], Belize [39], and India [40], for example. Particularly high F_IC _values were obtained for ophthalmological problems, tooth ache, kidney problems, and menstrual disorders (Table 3), indicating that the species that are traditionally used to treat these ailments are worth searching for bioactive compounds: *Berberis asiatica*, *Astilbe rivularis*, *Juniperus recurva*, *Swertia multicaulis, and Hippophae salicifolia*. The latter three species, as well as *Valeriana jatamansi*, are also of interest as they are traditionally used to treat three or four different ailment types (see Table 1).

### Bio-efficacy of medicinal plants

Empirical observations on the use of medicinal plants by the *Tamang *people of the Rasuwa district needed to be substantiated with phytochemical and pharmacological studies in order to corroborate their bio-efficacy. Such concerns were raised by ethnomedicinal studies carried out in Nepal, but few studies have provided the needed evidence [19,20]. Comparison of local uses and phytochemical/pharmacological properties for 30 medicinal plant species showed that traditional use was coherent with known phytochemical or pharmacological properties in 90% of the cases (Table 5).

Comparison of the information on traditional medicinal plant use in the Rasuwa district with ethnobotanical studies conducted in other areas of Nepal [15,18,20,41] shows similar results for many species. This is of significance because identical plant use by different people from different areas may be a reliable indication of curative properties. Like in other rural communities of Nepal [15,20,42,43], knowledge about traditional uses of medicinal plants is transferred from the household seniors and other elders. In many cases, this knowledge is transmitted orally, from generation to generation, and remains confined to a limited group of people [44]. Documentation efforts undertaken by Nepalese researchers in order to document traditional use of medicinal plants [13-15,20,45] should continue, especially as the results presented here place traditional and scientific knowledge on equal footing.

### Sustainable management and use of medicinal plants

The criteria used to identify priority medicinal plant species [32] are very practical and useful in the regional context and are highly reliable as they were synthesized by experts based on national and international data. The 16 priority species identified here are highly valued on national and international markets [46]. Importantly, 13 of the 16 species prioritized in the present study are also priority species identified by the central Government of Nepal, which recognized 30 medicinal plant species for promotion of commercial use and trade [32]. Therefore, it is important to consider these species to implement policy and to guide management authorities of the Rasuwa district for proper management and use of medicinal plants to benefit local people in their traditional healthcare delivery systems and income generation activities [6,47].

Unsustainable harvesting, over-exploitation and habitat degradation have been identified as major threats to the sustainability of medicinal plants in the district. The medicinal plants sector has the potential to achieve sustainability, given the availability of resources, people's willingness to participate in conservation programmes, and the priority given to the sector by the government and other organizations. Sustainable harvesting, effective domestication methods, community participatory management, and the provision of information, education and awareness programmes to the community are key strategies that can help optimize the benefits of the medicinal plants sector in Nepal [45].

## Conclusions

The *Tamang *people of the Rasuwa district of central Nepal possess rich ethnopharmacological knowledge and therefore use several medicinal plant species in their traditional healthcare delivery system. The striking coincidence between traditional plant use and scientifically-proven phytochemical and pharmacological properties shows that the traditional remedies are an important and effective part of indigenous healthcare systems in the district. However, published information on phytochemical and pharmacological properties are still limited for many plant species used in the district. Detailed phytochemical and pharmacological studies of traditionally-used medicinal plants is thus an important line of research to pursue, especially for species showing high informant consensus, like *Astilbe rivularis*, *Berberis asiatica*, *Hippophae salicifolia, Juniperus recurva*, and *Swertia multicaulis*. Medicinal plants provide huge opportunities for community development and livelihood improvement. However, local people are often deprived of the benefits from these resources [45]. Proper management of high-value and high-priority medicinal plants could serve as a sustainable income source for the communities. This would in turn help generate incentives for biodiversity conservation, thus ensuring the long-term availability of medicinal plants for indigenous and commercial uses.

## Competing interests

The authors declare that they have no competing interests.

## Authors' contributions

YU and SY carried out field research. EKB and KKS supervised the work. YU and HA analyzed the data and wrote the manuscript. All authors approved the final version of this manuscript.

## Supplementary Material

Additional file 1 **List of medicinal plants identified by *Tamang *people from the Chilime Village Development Committee of the Rasuwa district, Central Nepal**. Contains a list of the medicinal plants identified in the present study by the *Tamang *people from the Chilime Village Development Committee of the Rasuwa district, Central Nepal. Plants are sorted by scientific name. For each plant, family name, vernacular name(s), life form, part(s) used, uses, and mode(s) of use are provided. Click here for file
