# Supplementary material for: Indigenous use and bio-efficacy of medicinal plants in the Rasuwa District, Central Nepal
Source: J Ethnobiol Ethnomed. 2010 Jan 26;6:3. doi: 10.1186/1746-4269-6-3 (PMC2823594; doi:10.1186/1746-4269-6-3)
Supplement: Additional file 1 — List of medicinal plants identified by Tamang people from the Chilime Village Development Committee of the Rasuwa district, Central Nepal. Contains a list of the medicinal plants identified in the present study by the Tamang people from the Chilime Village Development Committee of the Rasuwa district, Central Nepal. Plants are sorted by scientific name. For each plant, family name, vernacular name(s), life form, part(s) used, uses, and mode(s) of use are provided. [file 1746-4269-6-3-S1.DOC]

**Additional file 1.** List of medicinal plants identified by *Tamang* people from the Chilime Village Development Committee of the Rasuwa district, Central Nepal.

|  | **Species name**  **FAMILY NAME** | **Vernacular name(s)*** | **Life form** | **Part(s) used** | **Uses** | **Mode(s) of use** |
| --- | --- | --- | --- | --- | --- | --- |
|  | *Abies spectabilis* (D. Don) Mirb.  PINACEAE | Gobre salla (Np),  Thasing (Tam),  Silver fir (Eng) | Tree | Leaf | Respiratory problems, cough. | Decoction used for bronchitis and cough. |
|  | *Aconitum ferox* Wall.ex Seringe  Ranunculaceae | Bikh (Np, Tam),  Aconite (Eng) | Herb | Root | Joint pain, orthopaedic problems. | Paste taken daily until recovery. |
|  | *Aconitum spicatum* (Bruhl) Stapf  Ranunculaceae | Bikh (Np),  Bingma (Tam),  Aconite (Eng) | Herb | Root | Fever, cuts and wounds, headache. | Decoction used for fever and headache, and paste applied to cuts and wounds. |
|  | *Acorus calamus* L.  Araceae | Bojho (Np),  Seda (Tam),  Sweet flag (Eng) | Herb | Rhizome | Flu (cough/cold, throat pain). | Small piece of rhizome chewed raw. |
|  | *Amaranthus spinosus* L.  Amaranthaceae | Naprukima (Tam) | Herb | Root | Cuts and wounds. | Paste applied. |
|  | *Anaphalis contorta* (D.Don) Hook.f.  Compositae | Buki phul (Np) | Herb | Flower, leaf | Chest pain, inner bleeding. | Infusion taken as tea. |
|  | *Artemisia indica* Willd Compositae | Titepati (Np),  Chyanchin, Surchent (Tam) | Herb | Leaf | Cuts and wounds. | Paste applied. |
|  | *Asparagus racemosus* Willd.  Liliaceae | Kurilo, Satawari (Np),  Komo (Tam) | Herb | Tubers | Fever, stomach ache, diarrhoea. | Paste taken daily until recovery. |
|  | *Astilbe rivularis* Buch.-Ham ex D.Don  Saxifragaceae | Thulo ausadhi (Np) | Herb | Root, leaf | Menstrual disorder. | Paste taken daily until recovery. |
|  | *Berberis asiatica* Roxb.ex DC  Berberidaceae | Chutro (Np),  Yamjuki Jungba (Tam), Barberry (Eng) | Shrub | Stem | Eye pain, rheumatisms. | Paste made of soft parts (cambium) applied for rheumatisms.  Paste made of inner yellow part (pith) used for eye problems. |
|  | *Bergenia ciliata* (Haw.) Sternb  Saxifragaceae | Pakhanved (Np),  Brasen (Tam),  Rock foil (Eng) | Herb | Whole plant | Indigestion, diarrhoea, dysentery, fever. | Juice. |
|  | *Bistorta affinis* (D. Don) Greene  POLYGONACEAE | Muakui (Tam) | Herb | Root, leaf | Diarrhoea and dysentery. | Paste drunk as tea in the morning. |
|  | *Cannabis sativa* L.  Cannabaceae | Ganja (Np),  Wang/Sima (Tam),  Hemp (Eng) | Herb | Stem, leaf | Stomach problems. | Paste is eaten. |
|  | *Cheilanthes albomarginata* Cl.  Pteridaceae | Rani sinka (Np),  Damkans (Tam) | Herb | Whole plant | Gastritis problems. | Juice. |
|  | *Cordyceps sinensis* (Berk) Sacc  Hypocreaceae | Yarsha gomba (Nep, Tam),  Cordyceps (Eng) | Fungal body | Whole plant | Tonic. | Juice taken after meal. |
|  | *Dactylorhiza hatagirea* (D.Don) Soo  Orchidaceae | Paanch aunle (Np),  Ongilakpa (Tam) | Herb | Tubers | Stomach problems, cuts and wounds. | Boiled and drunk for stomach problem. Paste applied to cuts and wounds. |
|  | *Delphinium himalayai* Munz  RANUNCULACEAE | Nirmansi (Np),  Bhongmar (Tam) | Herb | Root | Fever, headache, cough and cold. | Paste. |
|  | *Drynaria propinqua* (Wall.ex Mett.) J. Sm  Polypodiaceae | Nakahyapsing (Tam) | Herb | Rhizome | Fever. | Infusion taken every day . |
|  | *Entada rheedei* Spreng  LEGUMINOSAE | Pangram (Tam) | Herb | Whole plant | Pain relief of bone. | Paste taken until recovery. |
|  | *Ephedra gerardiana* Wall. Ex Stapf  Ephedraceae | Somlata (Np, Tam),  Ephedra (Eng) | Shrub | Stem | Asthma. | Tea in the morning. |
|  | *Eupatorium adenophorum* Spreng.  Compositae | Banmara (Np),  Kurum (Tam) | Shrub | Leaf | Cuts and wounds. | Juice applied to cuts and wounds. |
|  | *Fraxinus floribunda* Wall.  OLEACEAE | Kipsil (Tam),  Himalayan ash (Eng) | Tree | Bark | Body pain. | Infusion taken as tea. |
|  | *Fritillaria cirrhosa* D.Don  Liliaceae | Kaakoli (Np),  Tiru (Tam) | Herb | Whole plant | Gastritis/stomach disorder. | Juice taken until recovery. |
|  | *Geranium nepalense* Sweet  Gereniaceae | Gurije (Tam) | Herb | Root | Fever, cuts and wounds. | Paste taken with water. |
|  | *Hippophae salicifolia* D.Don  Elaegnaceae | Govo taru (Tam), Seabuckthorn (Eng) | Tree | Fruits | Diarrhoea, cough, menstrual disorder. | Juice taken as needed. |
|  | *Hippophae tibetana* Schlecht.  Elaegnaceae | Taru (Tam),  Seabuckthron (Eng) | Tree | Fruits | Stomach pain and diarrhoea. | Juice taken as needed. |
|  | *Juglans regia* L.  Juglandaceae | Okhar (Nep),  Himalayan walnut (Eng) | Tree | Fruits (Nuts) | Body fresh-up. | Tea made from inner black part. |
|  | *Juniperus recurva* Buch-Ham ex D.Don  Cupressaceae | Dhupi (Np),  Sukpa (Tam),  Juniper (Eng) | Tree | Fruit, leaf | Throat pain, kidney disorder, fever. | Paste taken. |
|  | *Lepisorus mehrae* Fras.-Jenk  Polypodiaceae | Tamda (Tam) | Herb | Rhizome | Dysentery. | Juice taken. |
|  | *Lindera neesiana* (Wall ex Nees) Kurz.  LAURACEAE | Kurum (Tam) | Tree | Fruits | Diarrhoea. | Juice taken. |
|  | *Lonicera myrtillus* Hook. f. &Thomson  CAPRIFOLIACEAE | Taktak (Tam) | Shrub | Root | Fever. | Paste taken until recovery. |
|  | *Lycopodium clavatum* L.  Lycopodiaceae | Nagbeli (Np) | Climber | Pollens | Cuts and wounds. | Paste applied. |
|  | *Lyonia ovalifolia* (Wall.) Drude  Ericaceae | Angeri (Np),  Tomasing (Tam) | Tree | Leaf | Wounds, scabies, boils. | Infusion applied. |
|  | *Mahonia napaulensis* DC.  Berberidaceae | Manechutro (Np),  Kyarpa (Tam) | Tree | Fruits | Dysentery. | Eaten raw. |
|  | *Myrica esculenta* Buch.-Ham. Ex D. Don  MYRICACEAE | Naming (Tam) | Tree | Bark | Heart problems. | Paste. |
|  | *Nardostachys grandiflora* DC.  Valerianaceae | Jatamansi (Np),  Pangne (Tam),  Spikenard (Eng) | Herb | Rhizome, leaf | Headache, high altitude sickness. | Juice. |
|  | *Neopicrorhiza scrophulariiflora* (Pennell) Hong  Scrophulariaceae | Kutki (Np),  Chungbarin (Tam) | Herb | Root | Recovery of whole body pain. | Juice. |
|  | *Onychium japonicum* (Thunb.) Kunze  Pteridaceae | Seto sinki (Np),  Timda (Tam) | Herb | Leaf, rhizome | Fever, skin problems. | Juice of rhizome taken for fever. Leaf and rhizome paste applied externally to treat skin problems. |
|  | *Paris polyphylla* Sm.  LILIACEAE | Satuwa (Np),  Kalchung (Tam),  Love apple (Eng) | Herb | Root | Fever, vomiting, worms. | Paste applied. |
|  | *Parmelia cirrhata* Fr.  Parmeliaceae | Jhyau (Np) | Lichen | Whole plant | Cuts and wounds. | Paste applied to cuts and wounds. |
|  | *Parmelia* sp.  PARMELIACEAE | Jhyau (Np) | Lichen | Whole plant | Cuts and wounds. | Paste applied to cuts and wounds. |
|  | *Phymatopteris quasidivaricata* (Hayat) Pich.Serm  Polypodiaceae | Nakapbyasing (Tam) | Herb | Root | Boils, chest pain, body pain. | Paste applied to boils, chest pain and body pain. |
|  | *Pieris formosa* (Wall.) D. Don  ERICACEAE | Prapra (Tam) | Shrub | Young shoot, Leaf | Headache. | Taken as tea. |
|  | *Potentilla fulgens* Wall. ex Hook.  Rosaceae | Bajradanti (Np),  Sangmen (Tam) | Herb | Root | Gastric, stomach pain. | Paste taken twice daily. |
|  | *Primula sikimmensis* Hook.f.  Primulaceae | Medoser (Np) | Herb | Whole plant | Fever, ulcer. | Boiled with water and taken. |
|  | *Rheum australe* D.Don  Polygonaceae | Padamchaal (Nep),  Chhurcha (Tam),  Himalayan rhubarb (Eng) | Herb | Root | Indigestion, diarrhoea, stomach ache, fever. | Juice. |
|  | *Rhodiola himalensis* (D. Don) Fu  CRASSULACEAE | Mahaguru (Np, Tam) | Herb | Root | Fever, stomach ache. | Paste taken twice a day. |
|  | *Rhododendron arboreum* Sm.  Ericaceae | Paremendo (Tam) | Tree | Flower | Tonic. | Juice. |
|  | *Rhododendron anthopogon* D.Don  Ericaceae | Sunpati (Nep),  Rhododendron (Eng) | Tree | Flower | Stomach ache. | Chewed raw. |
|  | *Rubia manjith* Roxb.ex Fleming  Rubiaceae | Majitho (Np),  Tiru (Tam),  Indian madder (Eng) | Herb | Root | Scabies/skin diseases. | Paste applied. |
|  | *Swertia chirayita* (Roxb. ex Fleming)  Gentianaceae | Chiraito (Np),  Timda (Tam),  Chiretta (Eng) | Herb | Whole plant | Fever, cold, headache. | Juice. |
|  | *Swertia multicaulis* D.Don  Gentianaceae | Sharmaguru (Np),  Chungbarin (Tam),  Chiretta (Eng) | Herb | Whole plant | Toothache  cough/cold, fever. | Paste applied on teeth. Juice drunk for fever, cough and cold. |
|  | *Taxus wallichiana* Zucc.  TAXACEAE | Siding (Tam),  Taxus (Eng) | Tree | Leaf | Respiratory problems. | Juice. |
|  | *Valeriana jatamansi* Jones  Valerianaceae | Sugandawal (Np),  Brasen (Tam),  Valerian (Eng) | Herb | Rhizome | Cuts and wounds, joint pain, Cough/cold, throat pain. | Paste applied. Rhizome chewed. |
|  | *Vitex negundo* L.  Labiatae | Simali (Np) | Shrub | Fruits | Worms. | Seed paste taken in the morning. |
|  | *Zanthoxylum armatum* DC.  Rutaceae | Timur (Np),  Yarma/Promo (Tam),  Prickly ash peeper, Nepali peeper (Eng), | Shrub | Fruits | Stomach ache and indigestion. | Crushed, pickled and eaten. |

*Np: Nepali name, Tam: *Tamang* name, Eng: English name.
